# Beyond Housing: Perceptions of Indirect Displacement, Displacement Risk, and Aging Precarity as Challenges to Aging in Place in Gentrifying Cities

**DOI:** 10.3390/ijerph16234633

**Published:** 2019-11-21

**Authors:** H. Shellae Versey, Serene Murad, Paul Willems, Mubarak Sanni

**Affiliations:** 1 Department of Psychology, Wesleyan University, Middletown, CT 06459, USA; pwillems@wesleyan.edu (P.W.); msanni@wesleyan.edu (M.S.); 2 Physicians for Human Rights, New York, NY 10018, USA; smurad@wesleyan.edu

**Keywords:** age-friendly cities, physical environment/space, urban ageing, gentrification, displacement, aging in place

## Abstract

Neighborhoods within age-friendly cities and communities are an important factor in shaping the everyday lives of older adults. Yet, less is known about how neighborhoods experiencing change influence the ability to age in place. One type of rapid neighborhood change occurring across major cities nationally and globally is gentrification, a process whereby the culture of an existing neighborhood changes through the influx of more affluent residents and businesses. Few studies have considered the impact of gentrification on older adults, who are among the most vulnerable to economic and social pressures that often accompany gentrification. The current study explores one consequence of gentrification, indirect displacement. While gentrification-induced displacement can refer to the physical (e.g., direct) displacement of residents moving out of a neighborhood due to rising housing costs, it also references the replacement of the unique character and social identity of a neighborhood (e.g., indirect displacement). We examine perceptions of the latter, characterized by perceived cultural shifts and housing concerns among adults aging in place in a gentrifying neighborhood in New York City. The implications of indirect displacement for displacement risk and aging precarity are discussed as potential threats to aging in place in age-friendly cities.

## 1. Introduction

The number of older adults living in cities and urban areas is expected to increase in the coming years [1,2,3]. As a result, guidelines developed by the World Health Organization aim to help cities and communities become more age-friendly [3,4,5,6]. One central concern in accommodating the growing population of older adults is the availability of and accessibility to affordable housing for adults who desire to remain rooted in their homes and communities [7]. Aging in place—or the ability to live in one’s own home and community safely, comfortably, and independently, regardless of age, race, income, or ability—is defined as the ideal by the majority of older adults and seniors [8,9,10]. However, there are challenges to achieving this goal for all those who desire to age in place that warrant further attention.

Aging in place can be difficult for lower-income adults with limited options for housing, particularly if neighborhood change results in more expensive rental and housing prices. Over the years, efforts to preserve affordable housing has resulted in the construction of Section 202 buildings. The Section 202 program helps expand the supply of affordable housing with supportive services for older adults, and allows them to live independently. Section 8 housing also provides housing assistance to moderate- and low-income households through the United States Department of Housing and Urban Development program [11]. However, in cities experiencing rapid turnover in many of its previous low-income neighborhoods due to gentrification, affordable housing has become increasingly scarce. In addition, the lack of affordable housing in gentrifying areas contributes to homelessness among older adults, which has received less attention in discussions about gentrification effects [12,13,14,15]. The current study explores these issues in New York City, a recognized age-friendly city that is undergoing gentrification, to better understand how housing scarcity within gentrifying neighborhoods presents obstacles to fully realizing an age-friendly agenda. Specifically, we aim to fill a gap in the literature about how gentrification can lead to various forms of displacement, highlighting implications for adults aging in place.

### 1.1. Literature Review

#### 1.1.1. Gentrification

The transformation of lower-income neighborhoods into higher-income neighborhoods is increasingly common, due to what has been called a “back-to-city” movement [16,17]. One result (or perhaps cause) of this movement is gentrification, typically characterized by affluent (often younger and White) residents moving into lower-income (often ethnically-minority) neighborhoods. What qualifies as gentrification has been heavily debated in the academic literature [18]. Gentrification is defined here as a large-scale housing market process in which more affluent residents move into an area of less affluence that results in changes of neighborhood character, sociodemographic composition, and higher prices for goods, services, and housing [17]. This process may lead to the direct and indirect displacement of lower-income residents [17,19,20,21]. While a growing body of research has given attention to the displaced (e.g., those forced to leave gentrifying neighborhoods), less is known about the people who remain behind [22,23]. Besides qualifying ‘what’ gentrification is, questions that probe ‘who’ is affected by it are often secondary. A third question that remains unclear is, what are the effects of gentrification for people who continue to live in these changing neighborhoods, particularly for older adults aging in place?

#### 1.1.2. Displacement: Direct and Indirect

Displacement can take several forms, direct and indirect. In one of the initial reports on neighborhood displacement, Grier and Grier [24] (p. 8) provide three criteria for defining displacement:

Displacement occurs when any household is forced to move from its residence by conditions which affect the dwelling or immediate surroundings, and which:

1. Are beyond the household’s reasonable ability to control or prevent;
2. Occur despite the household’s having met all conditions of occupancy; and
3. Make continued occupancy by that household impossible, hazardous, or unaffordable.

Therefore, when a household can no longer afford to remain in the same residential unit, residential displacement is a direct consequence (e.g., direct displacement). Direct displacement includes involuntary moves such as evictions, increased rent, and deliberate neglect of dwelling upkeep (by a landlord), all of which threaten a residents’ ability to stay put [25]. Subsequent reports note that displacement is the negative outcome of gentrification specifically, not other types of neighborhood upgrading [26]. As a result, affordable housing available to lower-income residents in gentrifying neighborhoods can become scarce, displacing longtime residents as the cost of living in the neighborhood increases.

**Figure 1:**
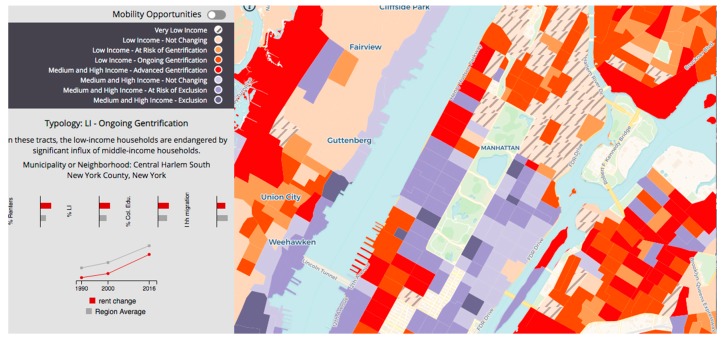
Source: Urban Displacement Project. Accessed October 19 at http://www.udpny.org/map.

In New York City, displacement pressures are severe. For example, Figure 1 indicates types of residential displacement across New York City, showing that a number of census tracts in Northern Manhattan, particularly in West and Central Harlem, are undergoing advanced gentrification, defined here as lower-income households experiencing a higher risk of displacement by higher-income households.

In some cases, mobility rates of lower-income people are similar for both gentrifying and non-gentrifying neighborhoods. Several researchers have interpreted this finding as evidence that displacement is not occurring [27,28,29]. However, an alternate explanation suggests that these patterns represent one type of mobility (e.g., out of neighborhood), and indicates that rates of mobility among lower-income residents are uniformly high across all types of neighborhoods. Rather than no displacement, these trends indicate that lower-income people move more frequently. Furthermore, there are other indirect ways in which individuals continuing to live in gentrifying neighborhoods can be displaced that are important to highlight [16,30].

Scholars suggest that a second consequence of gentrification is indirect displacement [31,32,33,34]. While less documented, indirect displacement may have a significant impact on older residents [35,36]. Indirect displacement describes a type of social displacement that occurs when incoming residents and/or businesses:

1. Drive housing demand that inflates costs that make buying or renting property unattainable for existing residents (also called *exclusionary* displacement) [37]; or
2. Change the feel, tastes, norms, and desires of an existing neighborhood, replacing the preferences or desires of existing residents (also called *cultural* displacement) [36,38].

Therefore, although remaining in place, residents staying put may still encounter displacement indirectly, evidenced by the increased cost of goods, services, housing, and a changing neighborhood culture [36,39]. Unlike direct displacement in which residents themselves relocate (voluntarily or involuntarily), indirect displacement is facilitated by moving the people, places, and structures *around* existing residents who are able to remain in the neighborhood, effectively recreating a new space designed to attract newer residents [20]. 

#### 1.1.3. Indirect Displacement and Aging in Place

Place is an important factor for how older adults see themselves within a community [9,40,41,42,43,44,45]. Research suggests that connectedness to public and semi-public spaces, community institutions, and landmarks strengthen social bonds, life satisfaction, and neighborhood identity, which are also significant for aging in place [16,39,46,47,48]. Since gentrification can change the ‘feel’ of a neighborhood by refashioning spaces in ways that cater to a selective few, neighborhoods can feel unwelcoming, even for longtime residents [5]. The establishment of new shops that feature expensive food or clothing, for example, convey implicit messages about who can and should enter these spaces [49]. In fact, a recent study finds that existing residents in gentrifying communities report feeling suddenly unwelcome in public spaces, playgrounds, and green spaces that should be accessible to everyone [50]. 

Broadly, if a place changes, feelings of displacement and/or exclusion can also be experienced, even if a person does not move. In this way, feeling unwelcomed in places that used to feel familiar may trigger insecurities and uncertainties about belonging, social exclusion, and the inability to feel ‘in-place’. For older adults especially, feeling ‘out of place’ can have implications for everyday life, perceived isolation, and overall well-being [5,50,51]. While almost no study has investigated links between gentrification, indirect displacement and belonging among older adults specifically, complementary research suggests that losses associated with direct displacement can be traumatic [52]. Atkinson [53] (p. 382) finds that descriptions of gentrification among the forcibly displaced include feelings of resentment, anger, “as well as a deeper sense of nostalgia for changing social relations and lost connections”. Studies have also shown that a sense of loss and grief can accompany residents who are forced to relocate to other places unexpectedly [32,47]. Whether the same loss is experienced when longtime residents manage to stay put in gentrifying neighborhoods remains a question.

#### 1.1.4. Opportunities to Expand Aging in Place Research

Age-friendly neighborhoods that offer a sense of safety, belonging, and inclusion can provide additional support for older adults, particularly for those who may have fewer friends and family members living close by. If place can be understood as a source of familiarity where one attributes meaning, identity, and importance, then the loss of places that residents consider ‘home’ should be explored further. Yet, little research in the United States has explored the importance of place, or how older adults can remain in place as they age.

In global and graying cities such as New York City, a limited affordable housing stock presents challenges for residents living in cities that have high levels of economic inequality [54]. For example, rent burden, or paying 30% or more of household income in rent, creates financial strain that contributes to precarity. Aging precarity represents instability in later life and usually stems from additional care and support needs, combined with limited disposable resources to accommodate such needs [55].

**Figure 2:**
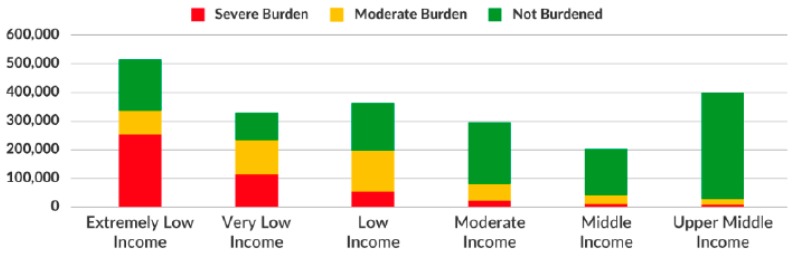
New York City renter households by level of rent burden by income group (2017). Note: Rent burden was calculated using Gross Rent Measure = Gross Rent Paid/Household Income.

In New York City, rent burden is highest among the most vulnerable–adults over the age of 65, as well as extremely low-income, and very low-income residents (see Table 1, Figure 2). Single, low-income adults over the age of 60 are the most vulnerable, subject to the highest rates of severe rent burden in the city. Yet research connecting aging in place to drivers of affordable housing for seniors, such as gentrification, is surprisingly scarce [5,23]. 

Therefore, one aim of this work is to understand how older adults describe the experience of living in a neighborhood that is changing. As many older adults express a continuing desire to age in place, it is important to understand how the ability to do so may be aided or compromised by neighborhood change. The traditional focus of aging in place research has been on physical features of the home that support growing old. This study aims to expand that focus to neighborhoods and communities using guidelines outlined by the Age-Friendly Agenda [56,57]. Since aging happens in a larger social and environmental context, realizing that agenda entails highlighting differential processes that may facilitate thriving, or simply surviving, during later years. The current study focuses on these issues within a gentrifying neighborhood in New York City to address the following research questions: What is the importance of neighborhood for adults aging in place? How do older adults perceive gentrification-induced change in their neighborhood, and do they perceive these changes as beneficial to older adults in the community?

## 2. Methods

### 2.1. Research Site: Central Harlem, New York City

**Figure 3:**
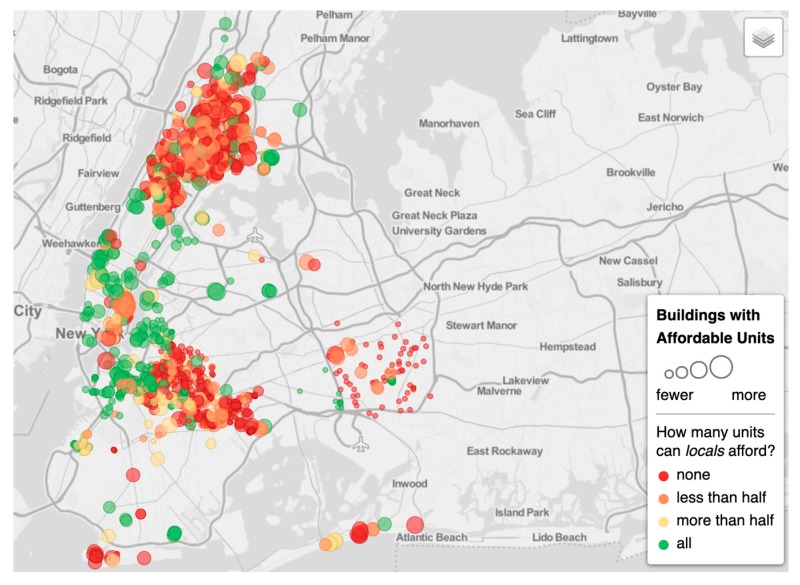
Buildings with affordable units for a typical median household, New York City (2018).

**Figure 4:**
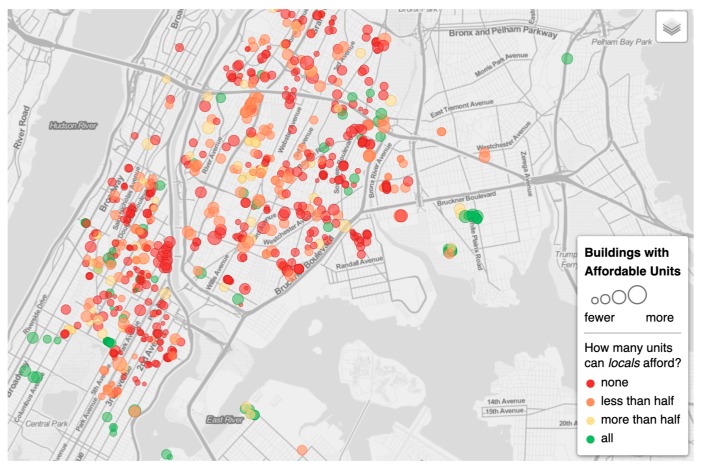
Buildings with affordable units for a typical median household, for the South Bronx and Northern Manhattan, including Central Harlem (2018). Source: Sam Raby’s data analysis for Curbed, Vox Media. Data sources: American Community Survey, New York Open Data, Housing and Urban Development Office of Policy Development and Research. Accessed September 30, 2019 at https://voxmedia.github.io/curbed-maps/HNY/

Within the five boroughs of New York City, parts of Manhattan have undergone extensive gentrification, particularly in Northern Manhattan, where rentals and home sales have set record highs [58,59]. For example, between 1996 and 2006, home prices increased by 270% in the Central Harlem neighborhood, decreasing the number of affordable housing units during the same period (see Table 2, Figure 2, Figure 3 and Figure 4). Central Harlem is a mixed-income community in the borough of Manhattan that is currently undergoing gentrification [59,60]. Central Harlem was selected for this study because it is a gentrifying neighborhood and home to a large, yet declining number of long-term and older African American residents. It is also a historical neighborhood and a culturally distinctive place among Black people and people of African descent–both foreign born and African Americans–across the diaspora generally [61,62].

Quantifying the impact of gentrification in numerical terms has numerous challenges, and secondary data analyses may underestimate various types of displacement that occur within and across gentrifying neighborhoods [35,63]. There are few studies that describe the experiences of aging residents in gentrifying areas, and fewer that engage topics of displacement, particularly among communities of color. Therefore, a qualitative approach is used to explore how older African American adults perceive markers of neighborhood change while staying put. All participants are aging in place, and have lived in the same community for the past decade with some level of independence. 

### 2.2. Participants and Demographic Characteristics

Participants were recruited from senior housing residential buildings and senior centers in the Central Harlem neighborhood. Nine focus groups with 98 African American men and women were conducted. Participants were required to have lived in Central Harlem for the past 10 years, be age 55 or older, and English-speaking. The minimum age limit of 55 was set to capture a wider variation of older adults, particularly those considered to be young-old [64,65,66].

The mean age of participants was 76 years old (SD = 1.64), with ages ranging from 55 to 92 years of age. Men and women were nearly equally represented, with women comprising 52% of participants. According to a short demographic survey administered prior to interviews, most respondents were single, widowed or divorced (82%). The average educational level was “some high school” (45.4%) or “graduated from high school” (16.8%). Eighty-seven percent were either retired (51%), unemployed (27.6%), or unable to work due to disability (8.4%). All respondents identified as Black/African American.

### 2.3. Participant Recruitment and Focus Group Facilitation

Participants were recruited through word of mouth from residential site managers, social workers, and paper flyers. Leading up to the first session, there was a four-week planning phase with all site coordinators regarding the scope and aims of the project. All (de-identified) data would be available to participants or coordinators following the conclusion of the project, if requested. This planning phase also included training of undergraduate students who would participate in the project.

At the first session, residents were invited to participate after a verbal explanation of the purpose of the study. Residents were informed that participation was voluntary, and all responses would be anonymously reported. Focus groups were held in the community space of each senior housing building, with the exception of one group that was held at a senior center. The research team was led by a trained and experienced African American female focus group facilitator. Three trained undergraduate students—one African American male, one African American female, and one European American female—served as research assistants for the study. Participants were encouraged to respond openly and honestly, regardless if thoughts and attitudes differed from other responses. Undergraduate students served as assistants and note-takers throughout the sessions.

Open-ended questions were posed in such a way to allow participants to be descriptive of the neighborhood, and all participants were encouraged to speak. With the consent of all participants, the discussion was taped and later transcribed verbatim by a third-party transcription service. Each focus group took approximately 60–90 min to complete and participants were paid $20 for their participation.

The focus group guide explored perceived neighborhood change as a function of gentrification. Sample questions included: What is most important about this neighborhood? Has the neighborhood changed in the past 10 to 15 years? If so, do you think of these changes as mostly positive or negative for the people who have been living here? The Human Subjects Research Institutional Review Board reviewed and approved the study, protocols, and materials (2016-0421-sversey-HCS). 

### 2.4. Data Analysis

Thematic analysis was used to identify and organize major themes from discussions [67]. Data analysts listened to audio recordings and read the transcripts several times, developing initial codes and synthesizing them into broader categories [68,69]. Data collected during each focus group were primarily analyzed for mentions of neighborhood importance and neighborhood change, whether it is occurring and to what extent, as well as potential benefits and challenges to aging in place. 

Inconsistencies in initial textual coding were discussed and resolved, and a second round of coding was conducted for accuracy and agreement (κ = 0.82) (95% CI, 0.500 to 0.886). Coding schemes were then categorized and discussed as a group, yielding three major themes—(1) neighborhood importance and identity; (2) perceived cultural displacement; and (3) housing concerns and financial precarity. Cultural displacement was defined according to conventional usage in the literature [48,70].

## 3. Results

### 3.1. Descriptive Analysis

The majority of respondents were renters (86.5%), with the highest percentage living in senior housing residences (HUD 202). The Housing and Urban Development (HUD) 202 program the only federal rental assistance program targeted specifically to older adults. In order to qualify for Section 202 housing, applicants must meet age and low-income requirements. Other participants lived in New York City Housing Authority buildings/public housing (15.6%) or rent-stabilized apartments (12.5%). 

Results are organized by major themes that emerged in response to study questions—neighborhood importance, neighborhood identity, perceived cultural displacement, housing, and financial precarity. Breaks between quotes indicate responses by different participants. In at least half of the conversations, the notion that the neighborhood had changed over time was spontaneously generated by participants themselves, allowing the facilitator to probe about specific changes, and ways in which these changes were perceived. In other cases (when change was not mentioned), the facilitator asked whether participants thought the neighborhood had changed any since they had been living there.

When participants mentioned neighborhood change or gentrification specifically, the facilitator asked for qualification regarding the meaning of ‘change’. Generally, changes referred to the number of new residents moving in, mostly White and higher income people, more African Americans moving out, increased housing/rental prices, the establishment of new businesses, and the provision of better community services, such as park improvement, more policing, and sanitation.

### 3.2. Neighborhood Importance and Identity 

When asked about what is ‘relevant’ or ‘important’ about the neighborhood, respondents overwhelmingly noted that it was “the people” that made Harlem special. When asked to elaborate, several participants recounted childhood stories growing up there, or referenced a time when they first moved to the neighborhood. Participants who had migrated to the neighborhood from other cities or boroughs discussed an attraction to the place, its vibrancy, its cultural familiarity, and the warmness of the people. A familiar refrain throughout the interviews was that the central identity of Harlem was a “mecca for Black people”, yet it was open and welcoming to all people.

#### 3.2.1. Neighborhood Identity

The notion that Harlem represented the people led to discussions that the neighborhood was changing into a ‘new’ version of itself, due primarily to gentrification. While some changes were welcomed (e.g., cleaner streets, better maintenance services) others voiced concern that “pushing out the people who made the neighborhood special” meant changing, or perhaps displacing, the culture of the place. For example, one respondent noted that even during the drug era of the 70s and 80s that plagued many urban neighborhoods, those involved in illicit activity were still part of the Harlem community and Harlem’s history for better or worse:

But it’s the people in Harlem that’s important. Now we’re not talking about the people that you see down on 123rd Street and Lexington Avenue (a place known for drug activity)…even though they’re still a part of us. They’re still a part of us and they just got lost. But they’re not the majority.

Others perceived that the people of Harlem built the neighborhood as it is known today (e.g., as a distinctive place to experience Black culture), and recent changes eroded a sense of what Harlem meant to the Black community.

One thing it does...it drives out the identity of what Harlem was.

I was born in Harlem, I said, “Okay, I’m going to die in Harlem.” You know, because regardless of all the crap that goes on in Harlem, these are my people. I love Harlem. And you know, as bad as it is, I still love Harlem.

Especially when you try to change the name [of Harlem], you erase all of what forefathers have done for us. That’s exactly what they’re trying to do. Not even the next generation, or the next, next generation is going to know what Harlem was.

Speaking of the Harlem Renaissance, we came up on 7th Avenue, on 138th Street. Remember the old Renaissance Ballroom? What is it now? It’s being built for luxury condos. And you know we can’t afford it, because everybody that I know on 132nd, 133rd, 134th Street—I was looking for them and they’re gone.

The final participant observes that not only is the Renaissance Ballroom (a historical structure) no longer standing, but the people who live in the surrounding blocks are also gone—an acknowledgement that if people make places, both have suffered as a result of gentrification. Responses suggest that a loss of place (e.g., displacement) is occurring even though the people themselves are remaining put.

#### 3.2.2. Neighborhood as ‘Home’

The second most frequently mentioned sentiment about the importance of Harlem (besides the people) was that it symbolized a sense of ‘home’. When probed about the meaning of ‘home’, respondents noted that they considered the neighborhood to be home. In addition to not wanting to leave the neighborhood, they felt secure in not having to leave, due to the financial housing subsidies provided to older residents that allowed them to remain put. Interestingly, while almost all respondents felt fortunate to have secured a unit within a senior housing building in which they were relatively protected from increasing rents, many participants expressed disappointment in living far from family members who were physically displaced due to not being able to afford to live in the same neighborhood. Perhaps as a result, several respondents expressed that the legacy of the neighborhood as being unique, distinct, and predominantly Black was in danger of being lost and should be preserved. 

### 3.3. Perceived Cultural Displacement and Black Space Erasure

Related fears about the erasure of people and historic places led to the expression of more general concerns about the future of Black people and Black spaces that cultivate inclusion, belonging, and ownership among Black people.

Harlem was described as one of the few places where Blacks could enjoy a sense of Black history and be welcomed by other Black people. One participant explains the transition from the way things were to a newer version of the neighborhood in terms of an “assault” on Black people, threatening a larger sense of community through displacement of the culture:

*It’s like an assault on black people. You know, you don’t want to say this, but it’s like a huge assault, forever, on black people. And if it’s not one thing, it’s another. It’s always to take us down or take us out altogether.*

*Another respondent highlights tensions between the economic boom in Harlem and cultural loss, suggesting that the “boom in diversity” may exclude Black residents already living in the neighborhood.*

*Just recently, it’s just been a boom in diversity here, of all nationalities. And the people that I growed up with in Harlem for the last 50 years, including now, they have really decreased in numbers. Its upsetting the culture balance. And our culture, as far as I’m concerned, our culture balance has been like drastically changed.*

*They taking our space. And we can’t live—we can’t go back to how it used to be.*

The idea that the neighborhood had changed in the past decade was unanimous among participants. Collectively, some participants viewed the neighborhood transition as change for a better community, enabling greater access to certain services. On the other hand, the majority of respondents viewed the changes as eroding a deeper sense of community and neighborhood identity, which were perceived to be at least equally important to, if not more important than, the improvement of amenities, some of which were viewed to accommodate incoming residents more than existing residents.

#### 3.3.1. Church Tourism

Participants also mentioned the unique aspects of the neighborhood were being marketed to others who lived elsewhere, in an attempt to draw them to Harlem. Several participants reported that churches were part of the problem, complicit in gentrification, catering to real estate developers and tourists. Generally, there was a sense of frustration that churches were not doing more to assist older people in the community who were vulnerable to displacement. 

Churches were also criticized for profiting from gentrification by selling church-owned property to developers and discontinuing social activities that kept the community connected. Larger churches were described as being in a position to use political and social influence to redirect, and even stop displacement efforts. However, participants perceived that the majority of pastors and church officials were preoccupied with financial gain.

*Churches have become tourist attractions. People come and fall in love with Harlem and the next thing you know, they move here! That’s how come most of the churches in Harlem got two services, because they couldn’t accommodate them in one service.*

*Now, if you go to any church in Harlem you’ll notice two-thirds of the churches now are filled with tourists. Yeah, they drop off two busloads of tourists here every Sunday.*

*Most of us, our churches are three-quarters tourists now. And that’s who’s coming here now–immigrants from Europe.*

Disagreement among participants regarding whether churches were helping or harming the community was expressed in the majority of interview groups. Some participants maintained that the church was still regarded as a place where community outreach and important charitable functions still took place. For example, most respondents who indicated that they were politically involved primarily participated in church-related efforts. 

#### 3.3.2. Outsider/Insider Tensions

Finally, respondents reported feeling ‘out of place’ when local businesses were replaced by more expensive retail options. Participants described the replacement of mom-and-pop businesses with new businesses that made Harlem more expensive and also made it feel less like a community.

*You don’t have any people, black people, owning stores anymore. It’s all Caucasian, different nationalities. We don’t have anything. We don’t have anything to say that the black people have a store. And all the stores that are opening is not for us.*

Most new businesses were perceived as providing services for newer residents. In addition, participants reported that competition with new businesses put a strain on mom-and-pop stores (primarily Black) that had been long-term establishments in the neighborhood.

*When you talk about economy, our people failed, not because they wanted to. They couldn’t get loans in the banks. Mom and Pop stores for blacks can’t get loans like Mom and Pop stores who are white.*

Besides the racial turnover in ownership of local businesses, several participants mentioned being treated “differently” when interacting with new shopkeepers.

*We went in there (Korean-owned beauty-supply store) and they were following us around. You know, we didn’t have that experience before. And my daughter said, “Mom, what’s this here?” She was really upset that it had changed like that. It had changed for me too because, you know, this is my home. This is my home, you know, this is where I grew up.*

This participant summarizes recurring tensions between residents and new, incoming businesses. If home is a place in which one feels safe and welcomed, negative neighbor relations may contribute to residents feeling alienated. When asked about other types of businesses in the neighborhood, such as restaurants and new eateries, participants expressed disinterest in entering such establishments, citing that they were “too expensive” and “not intended for them”. As one respondent noted:

*Well, we accept everybody in Harlem but we’re not accepted everywhere we go.*

On the other hand, some participants welcomed new businesses into the neighborhood as a sign that Harlem was improving. New supermarkets that provided more fresh fruits, vegetables and food options, for example, were considered amenities worth paying for. In some cases, these businesses and improved services were seen as markers of progress that strengthened the community.

*I have nothing against white folks moving in to my neighborhood, okay? Because my garbage is getting picked up better.*

While a few participants thought gentrification was positive since it brought selective improvements such as cleaner streets and better public services, others voiced frustration that services such as trash pick-up and community policing seemed to be a priority only when new residents moved in. Since these and other amenities were done in selective areas (i.e., mostly where newcomers lived) they were perceived as serving the needs of the community only when newcomers arrived. Interestingly, some participants expressed the idea that “White people complain more” to get things accomplished as one reason why some amenities had improved. 

Overall, participants reported that the neighborhood was important—as a physical ‘home’, but also as a place that Black people generally could call home due to its history and sense of being welcoming. In all nine focus groups, there were references that a cultural piece of Harlem had been lost, or was in danger of disappearing. Follow-up probes about precisely what has been lost yielded a variety of answers including a sense of history, the Black community, its people, and most of what has made it unique in the past. At the same time, participants across all sites were divided about whether these changes were entirely positive or negative.

### 3.4. Housing Concerns and Financial Precarity

#### 3.4.1. Housing Concerns

Respondents agreed that Harlem had become a more expensive place to live, and this was a frequent response to the question regarding whether the neighborhood had changed in the past decade. Although several participants agreed that they were not personally impacted by rising housing costs (those living in HUD Section 202 Housing), one participant argued that it was the lack of affordable housing in the neighborhood generally that pushed other longtime residents out, changing who was able to move in and thus, why the neighborhood felt less welcoming. One woman who had been unable to afford her previous rent before finding her current apartment commented:

*My apartment was also the same thing. I started out paying $500 and before I knew it they want $2500. So, it’s crazy. Every time you turn around they open up a new outside restaurant but people got no place to live.*

According to the same participant, rising rental costs are misaligned with the incomes of current residents, particularly seniors on limited budgets. 

#### 3.4.2. Housing Precarity

Increasing rents were mentioned as particularly troublesome for residents in the past, creating a cycle of living in multiple places for a period of time, and then moving when the rent was increased. While these moves would not qualify as displacement out of the neighborhood, the need to move multiple times within the same neighborhood represents a type of displacement pressure and housing precarity that is typically not captured in traditional displacement studies.

In addition, respondents mentioned that long waiting lists to get an apartment in senior buildings forced older renters out of the neighborhood who could not afford to wait. Participants reported waiting two to five years on average to be notified about an available unit. Both accounts reference a type of indirect displacement that ‘prices out’ would-be renters, who, as a function of rising rents and limited affordable housing stock, are no longer able to live within the neighborhood. 

Several participants suggested that the increasing rents were intentional, in order to move existing (Black) residents out, incentivize new (White) residents to move in, and encourage new development in the neighborhood.

Poor people can’t afford it. Therefore, in changing the neighborhood you’ll need more white people to sustain it.

*When I say housing, [I mean] the accessibility to affordable housing. Okay? So now, if you can afford it, you’re taking more of your income to pay rent, as opposed to saving and doing other things with it that you could have been doing.*

While the phrase ‘rent burden’ was not used in conversations, participants reported that much of their income was devoted to paying rent, leaving fewer resources for recreational or discretionary purposes. Similar examples of rent burden and housing strain were consistent across all study sites. 

#### 3.4.3. Homelessness

Conversations about being rent-burdened overlapped with concerns about financial security. Chief among housing-related concerns was the prospect of no longer being able to afford to live in the neighborhood, should a health emergency or some unexpected need arise. Ten participants across three separate groups independently introduced the notion that in such circumstances, they had known family members or acquaintances who had resorted to living in shelters. One participant disclosed that he had previously lived in a homeless shelter. Across several sites, respondents described a variety of financial situations that preceded un-homing friends or relatives, compromising their ability to stay housed.

Mod:      Where do you think people are going?
Participant P:  Shelters.
Participant K:  Sleeping on the street.
Participant L:  Homeless.
Participant C:  Yeah, sleeping on the street.
Mod:      Oh?
Participant K:  Yeah, because even the shelters are getting crowded now.
Participant P:   Right, they were crowded before, but they’re worse now.

At a different study site, one participant who disclosed having previously worked in the social service system in the city mentioned the hidden elder and working population that lives in homeless shelters, which she believed to be increasing.

The shelter population is ridiculous right now. It’s like it’s so many people in the shelters. And we’re talking working people, working people! Not they’re doing drugs on the street. They cannot pay their rent with the money they’re asking for rent.

Taken together, while participants felt affordable housing was an issue for the community, discussions focused on the more indirect effects of increasing rents that pushed residents to other precarious housing arrangements, still within the same neighborhood. The idea that Harlem was a more expensive place to live now, however, was only partially directed towards housing. Residents discussed how increased housing costs created additional spillover effects. 

#### 3.4.4. Gentrification and Financial Insecurity

The increased cost of living was considered the greatest threat to the ability to remain in the neighborhood. In other words, while participants were not *directly* displaced through eviction or relocation out of the neighborhood, the cost of living was cited as a major challenge to being able to age in place and enjoy a reasonable quality of life. For example, participants mentioned that although they were able to pay rent, they often had difficulty affording other necessities such as food and transportation, coinciding with other citywide changes in public benefits (e.g., reduction in food assistance programs and increases in MTA public transit fares) [71,72]. 

Several participants reported that rent consumed most of their monthly income. Residents who lived in public housing were concerned by increasing rents that would accompany privatization efforts headed by the New York City Housing Authority (NYCHA) and private interests (e.g., Rental Assistance Demonstration—RAD conversion). When asked whether or not privatizing public housing would be beneficial for older, and lower-income families, responses were mixed.

They want to clean it up. You know, that would be one way of making it safe and better for residents.

Yes, and they want the buildings. And they want the real estate—so now they’re pushing them [public housing residents] all to the Bronx or Long Island.

The last point related to “pushing low-income residents out” led to subsequent discussions about the impact of gentrification on outer boroughs, traveling longer distances to visit family, and less frequent visits from family.

#### 3.4.5. Summary of Findings

Overall, participants felt that their neighborhood was important to them and felt like home. Despite some benefits that accompanied neighborhood change, the majority of participants stated that the essence of the neighborhood was being displaced by newcomers. Church tourism and insider/outsider tensions were cited as visible markers of a changing neighborhood feel, while housing scarcity and financial pressures contributed to concerns about aging in place, and aging near family. 

## 4. Discussion

### 4.1. Indirect Pathways: Gentrification-Induced Displacement 

This study examined a sample of older Black adults’ experiences aging in place in a gentrifying neighborhood. Our findings highlight the social, financial and mental health consequences of neighborhood change for older adults—neighborhood identity, cultural displacement, housing, and financial precarity. These markers describe how older adults felt displaced despite remaining in the neighborhood, suggesting that there are indirect ways in which gentrification can engender displacement. These results are also notable considering that previous research has found little evidence of displacement in Central Harlem using quantitative indices. Nearly all respondents considered the neighborhood to be ‘home’ and wanted to remain in the neighborhood in hopes of aging in place. Indirect displacement has received less attention in previous research on gentrification; yet it may have several important consequences, particularly related to aging in place. 

In some ways, this research reflects what is known about the effects of gentrification, however, findings related to cultural displacement and older adults specifically illuminate new ideas about how residents understand displacement and relatedly, what makes place (e.g., placemaking). Lefebvre’s [73] idea that space is a social production permeates underlying narratives about gentrification and the terminology used to describe it. The language of renewal, revitalization, and reinvestment, for example, suggests a new valuation placed upon a previously devalued space. In fact, this study reveals that the Harlem neighborhood always held value, even in times of economic ruin, based on its cultural significance to the people living there. This set of realities has been called ‘Black placemaking’, to reflect the ways that Black people have created sites of cultural relevance, endurance, belonging, and resistance in spite of segregation, redlining, disinvestment, and neglect [74]. 

While this literature has not been linked to gentrification previously, the notion of placemaking extends knowledge about how neighborhoods function as places for how people see themselves in a larger context (e.g., place identity) [75,76]. While certainly not specific to Harlem, Black enclaves and residential communities are one way older residents might connect with a larger community, feel welcomed and appreciated [77]. In this way, Harlem is not simply a neighborhood, it is an example of Black placemaking, evidenced by participants’ reflections of how residents were able to transform what others deemed as disorder into authentic experiences, community, and spaces of inclusion and celebration [74,78]. Therefore, in the absence of being welcomed elsewhere, Black neighborhoods provide Black people an opportunity to carve out places where they satisfy the need to belong. 

Developing spaces that are inclusive of older residents is a central priority of age-friendly initiatives. However, our study finds that gentrification may increase the invisibility of aging adults by contributing to indirect and direct processes that erase people and places of significance. According to Kelley and colleagues [79] (p. 56) as cited by Buffel and Phillipson [5]:

Erasure is a concept used as a social critique of the ways certain groups of people are simply unseen in policy, research, or institutional practices. It is a form of social exclusion so embedded in the cultural assumptions of a society that the absence of these groups is not even recognized.

Future research may consider how to reconcile new development with preservation efforts in collaboration with the community to increase visibility for all residents. Public, residential, and commercial spaces should be examined further, since together, they shape the way neighborhoods are experienced. Policy that specifically targets older adults and aging in place is one way to include interests that have previously not been recognized. Expanded rent protections for low-income and extremely low-income residents, for example, can make communities accessible to groups with fewer resources. 

### 4.2. Precarious Aging in Place

A primary finding suggests that the ability to afford (and have access to) housing, goods, and services is important in order to remain rooted [23,80]. When new, more affluent residents move in, existing residents may benefit in some ways. Residents may also find it difficult to buy or rent property in the same neighborhood at the same rate, leading to housing precarity, a state of uncertainty and instability [81,82]. Aging precariously in place means that older adults are often managing multiple challenges tied to housing, such as housing-cost burden, financial insecurity, changes to mobility, and/or experiencing social isolation [83].

Lower-income and African American older adults are more likely to be housing cost-burdened than other groups, and are more likely restrict (or skip entirely) meals, food purchases, prescriptions, and transportation services in order to pay for rent [80]. Among older residents already facing severe financial challenges, making decisions between paying for food or housing may mean forgoing important primary or preventive medical care [50]. Housing instability was mentioned as a housing concern in which rising rents led to frequent moves and in some cases, homelessness. While homelessness among the working poor has received little attention in the larger conversation on gentrification, it was mentioned as a real fear (and reality) for several residents. Housing costs also contributed to what participants described as rent burden, leaving fewer resources for food or other expenses. Housing costs also complicated ways to receive and provide assistance to family members, many of whom were displaced to outer boroughs. 

### 4.3. Limitations

There are some limitations to this research worth noting. This study focuses on one neighborhood as a case study to illustrate the effects of gentrification. Therefore, the findings are not necessarily generalizable to other neighborhoods within New York City, or other age-friendly cities generally. Given this, however, we caution against oversimplifying the findings as being unique since similar trends have been found in other metropolitan areas in the United States, including Seattle, San Francisco, and Washington, DC. 

Second, although the focus of this study is older adults, it is likely that the issues raised in the current study are not specific to aging in place, but extend to other groups as well, including younger Black Americans and/or other racial-ethnic groups. Since all participants were required to speak English, there may be additional neighborhood dynamics among lower-income, non-English speaking adults that the current study was unable to observe. We do hope this research ignites dialogue and further discussion about the far-reaching implications of gentrification, cultural displacement, and precarity for all socially vulnerable groups.

### 4.4. Implications for Future Research

#### 4.4.1. Age-Friendly Communities in Gentrifying Cities?

A robust body of research shows that neighborhoods are increasingly important to support health and aging [84,85,86]. Meaningful social connections become important with age, since older adults are more likely to frame their daily activities and interpersonal interactions within their immediate neighborhoods [87,88]. When a neighborhood changes, new neighbors can create a less-inclusive social environment that can contribute to long-term residents feeling ‘pushed out’ of one’s own neighborhood. Therefore, understanding how gentrification reshapes networks, a sense of belonging, and ‘age-friendliness’ is important.

Our research finds several consistencies worth noting. One consistency is that participants do not want to lose the essence of their neighborhood, nor feel like strangers in it. Another consensus is that neighborhoods undergoing gentrification are more expensive to live in, and as a result, make some aspects of aging in place more difficult. A lack of affordable housing, the closure of longtime businesses, and feeling socially and financially insecure increased feelings of uncertainty among participants, potentially hurting, rather than helping older adults wanting to age in place. 

Future research on age-friendly cities should address the availability of affordable housing, as well as how housing scarcity contributes to secondary effects (e.g., homelessness) mentioned in the current study. Few studies have addressed homelessness as a byproduct of gentrification, and additional research will yield knowledge about how to ensure housing is accessible for all residents, particularly seniors. Similar to other national models that have developed pilot interventions for evaluating and measuring the collective impact of age-friendly community initiatives [89,90], future research should consider assessing indicators of elder displacement and homelessness as metrics of how well age-friendly cities and communities are meeting the needs of its constituents. 

#### 4.4.2. Mixed-Methods, Counter Mapping, and Policy Changes 

Improved metrics that use standard measures to document gentrification, displacement, and displacement risk would be useful in assessing the broad impact of gentrification on older and long-term residents. For example, the Anti-Eviction Mapping Project combines several mixed-method strategies including data visualization, data analysis, and oral histories to ‘counter map’ patterns of eviction and displacement across San Francisco and Alameda counties [91]. A similar strategy used by the Urban Displacement Project shows that over 33% of low-income households across a 31-county region in the New York metropolitan area live in low-income neighborhoods at risk of or already experiencing displacement and/or gentrification pressures, representing over 1.1 million low-income households [92]. Mapping techniques are tools that call political attention to high-risk areas and help ensure accountability and equity [93]. While some cities track indices of displacement risk, such as evictions, these audits are not consistently used. As one necessary feature of the Age-Friendly Cities and Communities project is to maintain sufficient and affordable housing, all age-friendly cities should be subject to a citywide displacement risk assessment. Collecting and aggregating city and countywide data will facilitate a review of residential shifts, highlight areas in most need of housing and aging services, and increase the visibility of low-income seniors at risk for housing displacement.

Broadly, additional research is needed to investigate the various forms of displacement that exist in cities and communities globally, particularly in understudied areas, such as global south cities, rural areas, and non-Western, non-European contexts [94]. Anti-displacement efforts typically aim to develop without displacement, consulting community members and establishing advocacy councils that work within the community to fine-tune development strategies. Advocates and city officials might review existing policies that encourage community-led investment initiatives to ensure that they are meeting the needs of older adults and enable existing residents to stay and benefit from renewal efforts. For example, interventions aimed at bridging residential divides can leverage churches, cultural institutions, grassroots planning, and city officials to develop strategies in which resident stakeholders communicate their needs to the broader community, re-envisioning what “revitalization” means [95].

Finally, older adults may be well-served by intergenerational community-building programs that could provide support and increase social engagement. Living in gentrifying neighborhoods increases stress burden for people of color, largely as a function of housing insecurity [96]. Therefore, opportunities to stay rooted and connected with other community members through homeownership, rental protections, and businesses opportunities may mitigate the effects of stress and ultimately stem the tide of displacement. 

## 5. Conclusions

Over a decade ago, Sabiyha Prince [61] wrote about changes in the “New Harlem”. Her interviews indicated that residents made a deliberate choice to live there because they “liked living around Black people”. One of her respondents commented, “I like living in a place where people say good morning to you and really care. I like the mix of elderly people. I wanted to be a part of our condition whether that be good or bad”. Another person noted, “Here you still get folks in the community walking up to you and saying, ‘Hi baby…how you doing?’ I want to hear that kind of language in my neighborhood, you know, people talking to me like I am welcome” [61] (p. 21). 

While much has changed since then, some things have not. Our findings support what previous research on Harlem has found—Harlem is a culturally significant place where Black people, old and young, want to live precisely *because* it is a Black neighborhood [60,62]. Almost all participants considered Harlem to be home, and wished to remain there. However, recent changes in the neighborhood were reported as threats to staying put. 

Growing older has its own challenges and invisibilities. Since gentrification has the potential to displace people and the cultural identity of a neighborhood, eroding a sense of belongingness, indirect displacement is worth highlighting in future studies on gentrification, aging, and neighborhoods. The notion that one can feel out-of-place while remaining in place requires additional theorization about how gentrification reconstructs place, place meanings, and the perception of age-friendliness. Furthermore, aging studies might consider the role of place attachment, neighborhood importance, and housing stability in shaping how older adults engage with others in changing neighborhoods. 

## Figures and Tables

**Table 1:** Renter households by rent-burden type and household characteristics, New York City (2017)

		Low Income Severely Burdened	Share of Low Income Severely Burdened	Share of All Households in Group
Total Households		420,798	100%	20%
Singles		202,644	48%	27%
	29 or younger	14,538	3%	19%
	30 to 59	79,194	19%	22%
	60 and older	100,912	26%	35%
Multiple Adults without Children		114,212	27%	15%
	29 or younger	17,685	4%	11%
	30 to 59	45,010	11%	11%
	60 and older	51,517	12%	24%
Single Parents		32,289	8%	26%
	29 or younger	4834	1%	30%
	30 to 59	25,926	6%	26%
	60 and older	1529	0%	20%
Multiple Adults with Children		71,654	17%	16%
	29 or younger	10,851	3%	20%
	30 to 59	54,673	13%	15%
	60 and older	6129	1%	19%

Source: Citizens Budget Commission staff analysis using U. S. Census Bureau data and New York City Housing and Vacancy Survey, 2017.

**Table 2:** Neighborhoods with the largest increases in home sale prices (1996–2006).

Neighborhood	Borough	% Increase
East Harlem	Manhattan (Upper)	499.6%
Morningside/Hamilton Heights	Manhattan (Upper)	398.7%
Washington Heights/Inwood	Manhattan (Upper)	333.4%
Central Harlem	Manhattan (Upper)	270.2%
Fort Greene/Brooklyn Heights	Brooklyn	261.5%
